# Serum uric acid to high‐density lipoprotein ratio as a novel indicator of inflammation is correlated with the presence and severity of metabolic syndrome: A large‐scale study

**DOI:** 10.1002/edm2.446

**Published:** 2023-08-21

**Authors:** Rana Kolahi Ahari, Amin Mansoori, Toktam Sahranavard, Monireh Sadat Miri, Sara Feizi, Habibollah Esmaily, Majid Ghayour‐Mobarhan

**Affiliations:** ^1^ International UNESCO Center for Health‐Related Basic Sciences and Human Nutrition Mashhad University of Medical Sciences Mashhad Iran; ^2^ Department of Biostatistics, School of Health Mashhad University of Medical Sciences Mashhad Iran; ^3^ Department of Biology, Faculty of Sciences, Mashhad Branch Islamic Azad University Mashhad Iran

**Keywords:** high‐density lipoprotein, metabolic syndrome, UHR, uric acid

## Abstract

**Introduction:**

We investigated the association of serum uric acid to high‐density lipoprotein ratio (UHR) with the presence and severity of metabolic syndrome (MetS) among MASHAD cohort participants.

**Methods:**

In this cross‐sectional study, according to International Diabetes Federation criteria, the cohort participants were divided into MetS (+) and MetS (−) groups. MetS (+) were classified into Group 1 (those with 3 MetS criteria), Group 2 (those with 4 MetS criteria) and Group 3 (those with 5 MetS criteria). UHR was compared among the groups.

**Results:**

Data related to 9637 subjects including 3824 MetS (+) and 5813 MetS (−) were analysed. The mean UHR was significantly higher (*p* < .001) in the MetS (+) group compared with the MetS (−) group. UHR increased as the MetS severity increased (*p* < .001). ROC analysis revealed that UHR greater than 9.5% has 89.07% sensitivity and 77.03% specificity in differentiating MetS (−) from MetS (+) subjects.

**Conclusion:**

Among MASHAD cohort study participants, a significant association between UHR and MetS was found. Furthermore, there is an increase in UHR as the severity of MetS increases. Registration number of MASHAD cohort study: 85134.

## Graphical abstract

(1) UHR is (significantly) higher in patients with MetS compared to those without MetS. (2) UHR increased as the MetS severity increases. (3) Serum UA is (significantly) higher in patients with MetS compared to those without MetS. (4) Serum UA increased as the MetS severity increases.

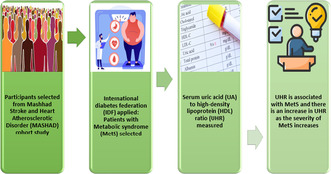

## 1 INTRODUCTION

Metabolic syndrome (MetS) is defined as a cluster of symptoms such as hyperglycaemia/insulin resistance, obesity, hypertension (HTN) and dyslipidaemia and it is directly linked to a higher risk of developing atherosclerotic cardiovascular disease (CVD), diabetes mellitus (DM) and all‐cause mortality.^1^ The prevalence of MetS began to increase globally due to obesity epidemics. It has been reported that obesity‐induced inflammatory processes may lead to a state of chronic low‐grade inflammation, which plays an important role in the development of insulin resistance that triggers the associated MetS comorbidities such as atherosclerosis, HTN, dyslipidaemia, hyperglycaemia and prothrombotic state.^2^, ^3^

Moreover, uric acid (UA) as the end product of endogenous and exogenous purine metabolism by xanthine oxidase, causes insulin resistance and atherosclerosis by platelet dysfunction, reducing the production of nitric oxide, endothelial dysfunction and oxidative metabolism. Studies showed that increased insulin secretion increases serum UA levels due to a decline in renal function and serum UA concentration is independently correlated with MetS risk factors, HTN, kidney disease and cardiovascular events.^4^, ^5^ UA can also induce inflammatory and oxidative changes in adipocytes.^6^, ^7^ On the contrary, high‐density lipoprotein (HDL) known as ‘good’ cholesterol is a cardioprotective biomarker, which removes excess cholesterol from peripheral tissues and transports it to the liver; for HDL, a spectrum of antiapoptotic, antithrombotic, anti‐inflammatory and antioxidative activities have been shown.^8^ A low concentration of HDL is identified as an independent predictor of CVD risk. On the contrary, it was discovered that depending on the presence or absence of inflammation, HDL may act both as an anti‐inflammatory or pro‐inflammatory factor for multiple metabolic disorders. Indeed, in addition to the quantity of HDL, the quality of HDL particles appears to be crucial and should be considered.^9^, ^10^, ^11^, ^12^ Investigations suggest that combination of HDL and serum UA may be a better predictor of CVD morbidity and mortality than either alone.^11^ Recently, serum UA to HDL ratio (UHR) has been reported as a novel inflammatory and metabolic marker, which increases in inflammatory conditions such as steatohepatitis and Hashimoto's thyroiditis.^13^ The predictive value of UHR has also been validated in DM and MetS.^14^, ^15^ However, whether UHR is associated with MetS severity remains unclear.

Since the *prevalence of hyperuricaemia* and MetS has been *increasing* not only in advanced *countries* but also in developing *countries*,^16^ *the early diagnosis is a crucial issue*. Also, due to both anti‐inflammatory and pro‐inflammatory properties of total HDL and since few studies assessed the relationship between UHR as a novel and easy‐to‐assess metabolic marker and MetS in a large‐scale population so far, the present study intended to compare the UHR between MetS patients and non‐MetS ones enrolled in MASHAD cohort study^17^ and examine correlations between UHR and of MetS severity.

## 2 METHOD

### 2.1 Study design and population

The target population was recruited from the Mashhad Stroke and Heart Atherosclerotic Disorder (MASHAD) study, a 10‐year prospective cohort from Mashhad city, northeast of Iran. The methodology and sampling details have been published elsewhere.^17^ In brief, 9704 individuals aged 35–65 years were recruited using stratified cluster random sampling technique. The socio‐demographic, hematologic, anthropometric and biochemical data were collected. Those whose biochemical data was not available were excluded from the present study.

In our study, MetS is defined based on the International Diabetes Federation (IDF).^18^ According to the IDF definition, someone has MetS if she or he has central obesity (≥94 cm for men and ≥80 cm for women) plus two or more of the following four factors:

1. Raised concentration of triglycerides (TGs): ≥150 mg/dL (1.7 mmol/L) or receives specific treatment for this lipid abnormality;
2. Reduced concentration of HDL cholesterol: <40 mg/dL (1.03 mmol/L) in men and <50 mg/dL (1.29 mmol/L) in women or receives specific treatment for this lipid abnormality;
3. Raised fasting plasma glucose concentration: ≥100 mg/dL (5.6 mmol/L) or previously diagnosed with DMII;
4. Raised blood pressure: systolic blood pressure (SBP) ≥130 mmHg or diastolic blood pressure (DBP) ≥85 mmHg or receives treatment for previously diagnosed HTN.

**FIGURE 1:**
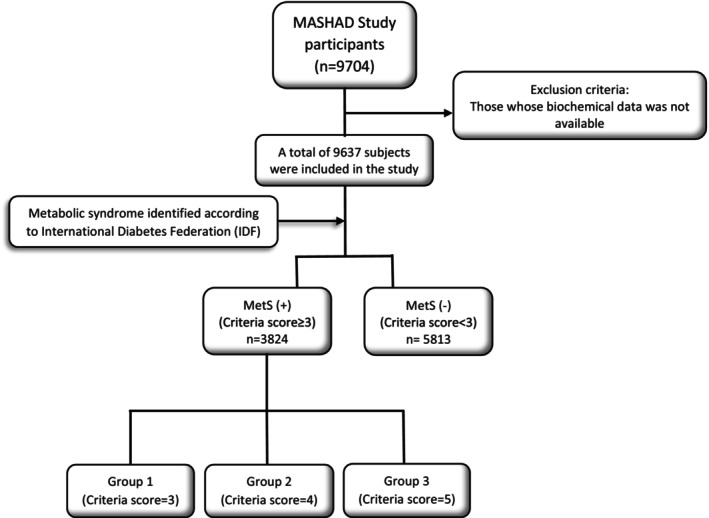
Flowchart of the present study participants and grouping.

Then, subjects classified as MetS (+) group were divided into three subgroups based on the number of MetS criteria they had as follows: group 1 (patients with three MetS criteria), group 2 (patients with four MetS criteria) and group 3 (patients with five MetS criteria). Rest of the population was selected as MetS (−) group. The flowchart of current study design outlined in Figure 1.

### 2.2 Ethical consideration

All participants included in the study were informed about the study, and signed a written informed consent form before inclusion. The study was approved by the Ethic Committee of Mashhad University of Medical Sciences.

### 2.3 Anthropometric measurements

Anthropometric measurements including height, weight and waist circumference (WC) were obtained. Height was measured by a standard wall height meter. Weight was measured by an analogue scale, which was kept on a firm horizontal surface. WC was measured with a non‐stretchable fibre measuring tape. The body mass index (BMI) was calculated as the weight (kg) divided by the square of the height (m).

### 2.4 Blood pressure

Participants were requested to relax for at least 15 min. Then, blood pressure of the right arm was measured and repeated after 10 and 20 min (a total of three times). If taking the blood pressure from the right arm was not successful, the left arm was considered. The mean of the three measurements was recorded as the participant's final blood pressure.

### 2.5 Biochemical parameters

In our laboratory unit, a 20‐mL blood sample was taken from each participant. Blood samples were taken between 8 and 10 a.m. by venipuncture of an antecubital vein after 14 h of overnight fasting. According to standard protocol, samples were collected in vacuum tubes (20 mL) from subjects in a sitting position and immediately centrifuged at room temperature to separate the plasma and serum into six aliquots (0.5 mL) and then sent to the MASHAD study biobank. BT3000 biochemical analyser was used for determination of biochemical parameters levels including: fasting blood glucose (FBG), UA, high‐sensitivity C‐reactive protein (hs‐CRP), Low‐density lipoprotein (LDL), total cholesterol (TC), TGs and HDL. All devices were daily checked by a laboratory technician. UHR was calculated as the ratio of serum UA (mg/dL) to HDL (mg/dL).

### 2.6 Statistical analysis and model building

To describe the quantitative and qualitative variables, mean ± SD and frequency (%) are reported, respectively. The Chi‐squared test and Fisher's exact test were applied to measure the association between categorical variables. Also, the mean of quantitative variables between the MetS (+) and MetS (−) groups were compared by independent *t* test. In addition, logistic regression (LR) algorithm was used to analyse data. In fact, we applied the algorithm to deduce the association between MetS and related factors. All analysis was performed using SPSS version 22 (IBM Corp.).

### 2.7 Logistic regression (LR) modelling

LR is a popular model to evaluate the relationship between various predictor variables (either categorical or continuous) and binary outcomes in medicine, public health, etc.^19^, ^20^, ^21^, ^22^, ^23^

Let $Y_{i}$ denotes the response variable and takes the values of 0 or 1 depending on whether response occurs or not. Also, X be vectors of covariates associated with response variable, β is the corresponding vectors of regression coefficients. So, the association between the covariates and binary response variable can be investigated as follows:

$\log\mathrm{it}\left\{E\left(Y_{i}\right)\right\}=\log\mathrm{it}\{\mathrm{Pr}\left(Y_{i}=1|X,\beta \right)=\beta^{T}X$

## 3 RESULTS

A total of 9704 subjects were enrolled to the MASHAD cohort study. After applying exclusion criteria, data related to 9637 eligible patients were analysed (3824 with MetS and 5813 without MetS) (Figure 1). Comparisons of the basic characteristics of the MetS (+) and MetS (−) groups are shown in Table 1. Mean ages of MetS (+) and MetS (−) patients were 46.90 ± 8.18 and 50.01 ± 7.98 years, respectively, indicated a significant difference (*p* < .001). In MetS (+) group, 1184 (30.96%) were male and 2640 (69.04%) were female and in the MetS (−) group, 2663 (45.81%) were male and 3150 (54.19%) were female. There was a significant difference between the two groups in terms of gender (*p* < .001). There was significant difference between the two groups in terms of BMI, WC, SBP, DBP, hs‐CRP, TC, LDL, TGs and FBG as well (*p* < .001). On the contrary, smoking status was not statistically significantly different between the groups (*p* = .53). The mean HDL level in the MetS (+) group was significantly lower than the MetS (−) group (39.68 ± 8.32 vs. 44.92 ± 10.37 mg/dL, respectively, *p* < .001). The mean UA level in the MetS (+) and MetS (−) group was 4.89 ± 1.41 and 4.49 ± 1.36 mg/dL, respectively (*p* < .001). Accordingly, MetS (+) group had a significantly higher UHR compared to the MetS (−) group (0.13 ± 0.05 vs. 0.11 ± 0.04 mg/dL, respectively, *p* < .001).

**TABLE 1:** Baseline and clinical characteristics of study population from Mashhad Stroke and Heart Atherosclerotic Disorder (MASHAD) study.

Characteristics	MetS (−) (*n* = 5813, 60.32%)	MetS (+) (*n* = 3824, 39.68%)	*p*‐Value*
Sex			
Female	3150 (54.19%)	2640 (69.04%)	<.001
Male	2663 (45.81%)	1184 (30.96%)	
Smoking status			
Smoker	1270 (21.84%)	798 (20.86%)	.53
Non‐smoker	3990 (68.63%)	2628 (68.72%)	
Ex‐smoker	553 (9.51%)	398 (10.40%)	
Diabetes			
Diabetic	392 (6.81%)	965 (25.41%)	<.001
Non‐diabetic	5361 (93.19%)	2832 (74.58%)	
HTN			
HTN+	997 (17.18%)	2015 (52.92%)	<.001
HTN−	4807 (82.82%)	1793 (47.08%)	
Age (year)	46.90 ± 8.18	50.01 ± 7.98	<.001
BMI	26.48 ± 4.51	29.99 ± 4.26	<.001
Cholesterol (mg/dL)	185.92 ± 36.54	199.55 ± 41.49	<.001
LDL (mg/dL)	115.11 ± 33.01	118.78 ± 38.27	<.001
TG (mg/dL)	111.38 ± 61.88	190.03 ± 109.86	<.001
HDL (mg/dL)	44.92 ± 10.37	39.68 ± 8.32	<.001
Glucose (mg/dL)	83.66 ± 26.64	106.38 ± 50.06	<.001
SBP (mmHg)	115.97 ± 15.33	130.56 ± 19.47	<.001
DBP (mmHg)	75.86 ± 9.92	83.99 ± 11.34	<.001
Uric acid (mg/dL)	4.49 ± 1.36	4.89 ± 1.41	<.001
UHR (mg/dL)	0.11 ± 0.04	0.13 ± 0.05	<.001
WC (cm)	91.21 ± 11.71	101.24 ± 9.84	<.001
hs‐CRP (mg/dL)	3.69 ± 8.50	4.85 ± 9.24	<.001

Abbreviations: BMI, body mass index; DBP, diastolic blood pressure; HDL, high‐density lipoprotein; HTN, hypertension; hs‐CRP, high‐sensitive C‐reactive protein; LDL, low‐density lipoprotein; SBP, *systolic* blood pressure; TG, triglyceride; UHR, uric acid to HDL ratio; WC, waist circumference.

* *p*‐value is computed based on *t*‐tests for continuous data and Chi‐squared test for categorical data.

Subgroup analysis demonstrated that in the MetS (+) group, there was a positive relationship between increasing number of MetS components (i.e. the severity of MetS) and serum UA levels (4.81 ± 1.36 vs. 5.01 ± 1.46 vs. 5.09 ± 1.53, respectively, *p* < .001) as well as hs‐CRP levels (4.47 ± 8.71 vs. 5.40 ± 10.39 vs. 5.50 ± 8.11, respectively, *p* < .001). There was no significant difference between the group 2 and 3 in terms of UA (Table 2). HDL was significantly decreased when the MetS severity increased (40.62 ± 8.62 vs. 38.54 ± 7.87 vs. 37.32 ± 6.62, respectively, *p* < .001). In addition, UHR was detected to be significantly lower in the MetS (−) group compared to the group 1, 2 and 3 (0.11 ± 0.04 vs. 12 ± 0.04, 0.13 ± 0.05 and 0.14 ± 0.05, respectively, and *p* < .001). Group 1 had significantly lower UHR than group 2 and 3 (*p* < .001), whereas there was no significant difference between the group 2 and 3 (*p* = .33).

**TABLE 2:** Comparison of group 1, 2 and 3 of MetS (+) population in terms of UHR, UA, HDL and hs‐CRP.

Variables	Group 1	Group 2	Group 3	*p*‐Value*
UHR	0.12 ± 0.04	0.13 ± 0.05***	0.14 ± 0.05***	<.001
UA	4.81 ± 1.36	5.01 ± 1.46^###^	5.09 ± 1.53^###^	<.001
HDL	40.62 ± 8.62	38.54 ± 7.87	37.32 ± 6.62	<.001
Hs‐CRP	4.47 ± 8.71	5.40 ± 10.39	5.50 ± 8.11	<.001

Abbreviations: HDL, high‐density lipoprotein; hs‐CRP, high‐sensitive C‐reactive protein; UA, uric acid.

* Used One way Anova test (variance analysis).

*** Group 2 and 3 were significantly different from Group 1 (*p*‐value < .001).

^###^ Group 2 and 3 were significantly different from Group 1 (*p*‐value < 0.001).

LR technique was used to investigate the relationship between predictors and binary response variables (MetS [+] and MetS [−]). So, the main objective was to anticipate MetS using the LR model and to determine MetS associated factors.

### 3.1 LR model

**FIGURE 2:**
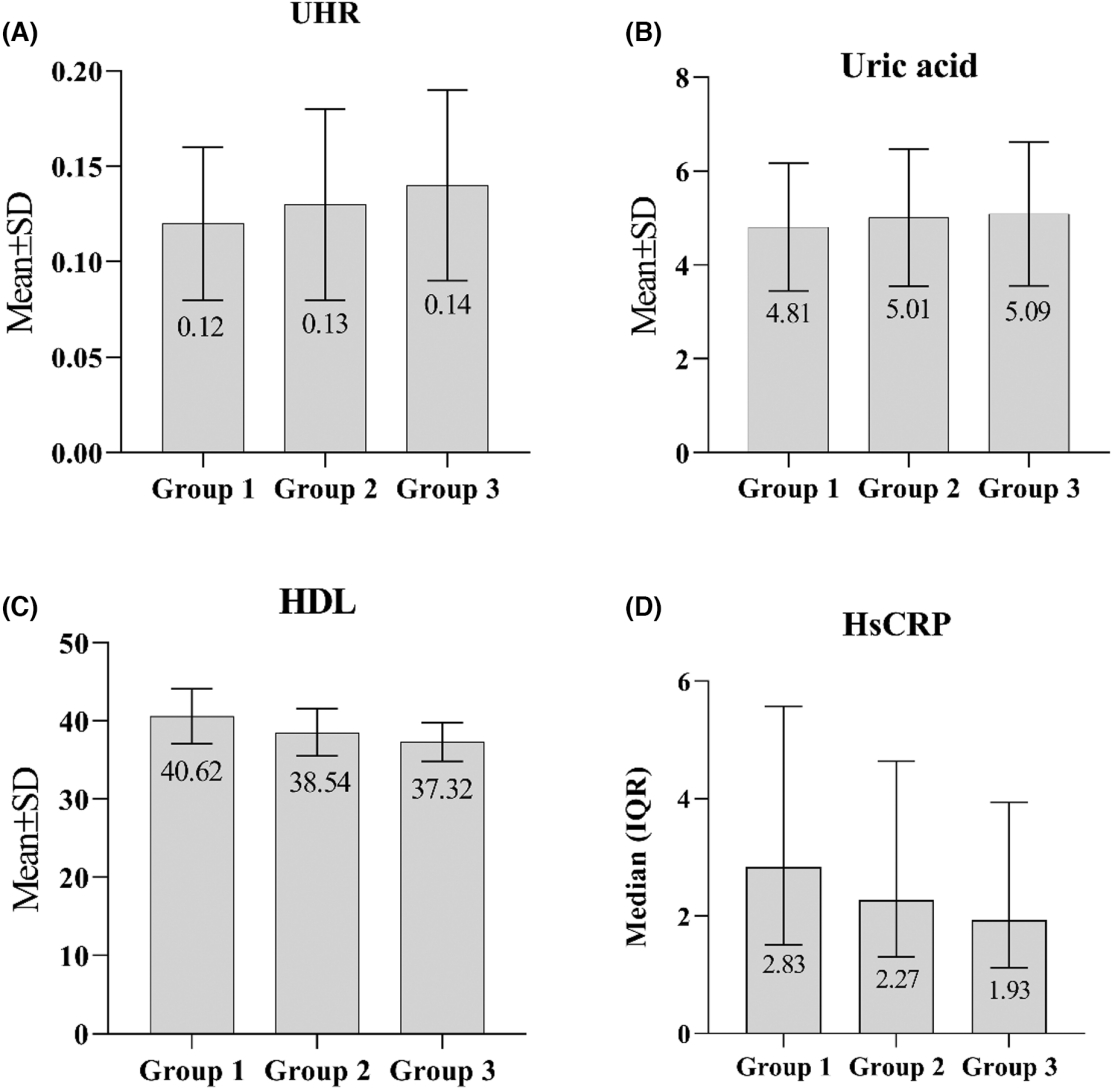
Comparison between UHR (A), UA (B), HDL (C) and hs‐CRP (D) and group 1, 2 and 3 of MetS (+) population. hs‐CRP, high‐sensitive C‐reactive protein; HDL, high‐density lipoprotein; UA, uric acid; UHR, uric acid to HDL ratio.

**FIGURE 3:**
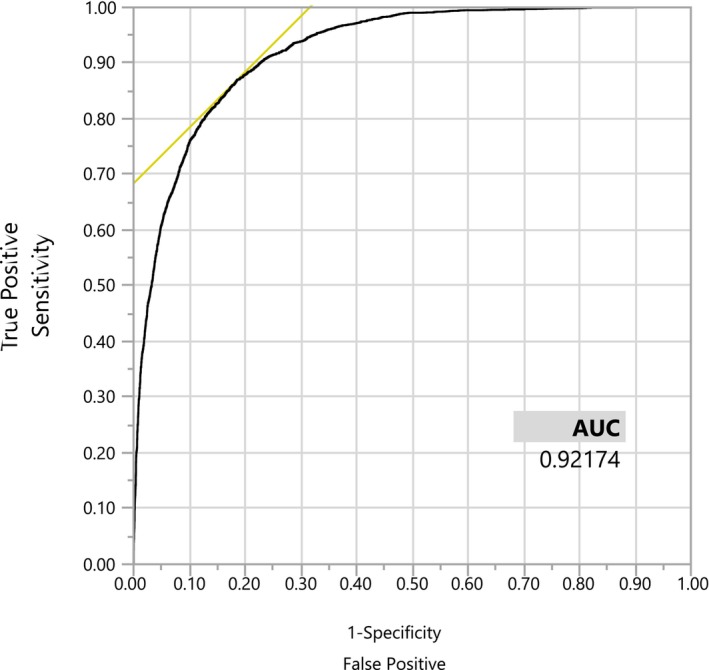
ROC curve of UHR in determining MetS. ROC, receiver operating characteristic; UHR, uric acid to HDL ratio.

**FIGURE 4:**
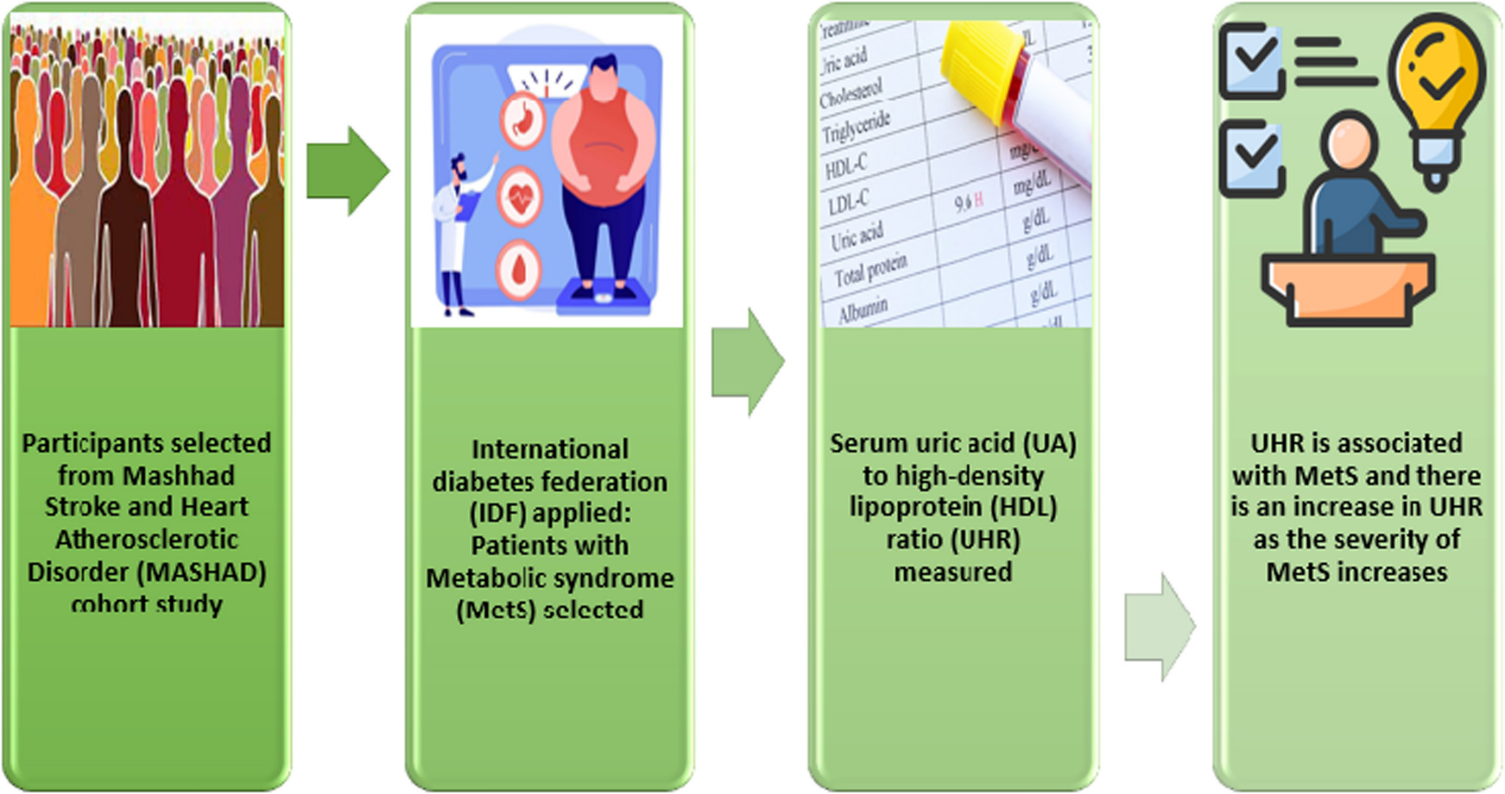
The primary findings of present study.

Results from the multiple LR model revealed that variables including sex, TG, WC, HDL, glucose, SBP, DBP, HTN, UA, TC, DM and UHR were significantly associated with of the presence of MetS (*p* < .002) (Table 3). In other words, our findings after adjusting the effect of other variables in the model, presented that the odds of having MetS in males is 0.14 times than that of females (*p* < .001). Also, after adjusting the effect of other variables for each increasing in UA, the odds of having MetS raised by 31% (*p* < .001) and for a 10‐unit increase in UHR, the odds of having MetS decreased by 54% (*p* < .001). Among the analysed variables, UHR (OR 0.55; 95% CI 0.38–0.79), UA (OR 1.31; 95% CI 1.18–1.47) and HTN (OR 2.01; 95% CI 1.70–2.37) had the greatest associations with MetS. In addition, Figure 2 shows the comparison between UHR, UA, HDL and hs‐CRP and group 1, 2 and 3 of MetS (+) population. Moreover, in order to determine the sensitivity and specificity of UHR, receiver operating characteristic (ROC) curve analysis was performed. The optimum cutoff was 9.5% with sensitivity 89.07%, specificity 77.03% and accuracy 84.30% (area under ROC [AUC] 92.17%) (Figure 3). We summarize the concept of the paper in graphical abstract (Figure 4).

**TABLE 3:** The results of multiple LR model for the MetS (+) group.

Source	Longworth	Odds ratio (95 %CI)	*p*‐Value
Sex (Female) Male	158.128	0.14 (0.12, 0.16)	<0.001
TG	148.778	1.01 (1.01, 1.02)	<.001
WC	140.938	1.07 (1.06, 1.08)	<.001
HDL	65.793	0.87 (0.86, 0.88)	<.001
Glucose	34.711	1.01 (1.01, 1.02)	<.001
SBP	20.559	1.03 (1.02, 1.03)	<.001
DBP	20.048	1.04 (1.03, 1.05)	<.001
HTN (HTN+)	15.603	2.01 (1.70, 2.37)	<.001
Uric acid	5.631	1.31 (1.18, 1.47)	<.001
Cholesterol	5.009	1.004 (1.003, 1.006)	<.001
UHR	2.723	0.55 (0.38, 0.79)	.001

Abbreviations: DBP, diastolic blood pressure; HDL, high‐density lipoprotein; HTN, hypertension; SBP, *systolic* blood pressure; TG, triglyceride; UHR, uric acid to HDL ratio; WC, waist circumference.

## 4 DISCUSSION

To the best of our knowledge, this is the first study conducted on possible association between UHR and severity of MetS in a large population. Our study showed that UHR was significantly higher in the MetS (+) group compared to the MetS (−) population. Subgroup analysis showed a correlation between the increases in the number of MetS criteria along with the increase in the UHR, serum UA and hs‐CRP.

MetS is a tremendously growing public health problem, which is comprised of interconnected and heterogeneous metabolic origin risk factors that appear to be directly promoting the development of type 2 DM, adverse cardiovascular events and all‐cause mortality.^24^ MetS induces low‐grade chronic systemic inflammation in the circulation and peripheral metabolic tissues, defined as ‘metabolic inflammation’, which is different from classical inflammation.^25^, ^26^ Numerous studies confirmed that CRP as a conventional marker of systemic inflammation is correlated with the number of MetS components (i.e. MetS severity) and a higher CRP level was associated with an increased incidence of cardiovascular events.^27^ In accordance with previous studies, our study showed that serum hs‐CPR level was significantly higher in the MetS (+) group and it was also significantly increased with the number of MetS components. Nevertheless, we found no statistically significant difference in term of smoking status between the MetS (+) and MetS (−) groups. A study by Daniel et al. reported that not former smokers had significantly greater insulin resistance compared to non‐smokers in a native American population.^28^ Hughes et al. reported that fasting insulin levels did not significantly differ between smokers and non‐smokers in a random sample of males aged 30–69 years from the general population of Singapore.^29^ This could be in agreement with the finding that smoking less than 10 cigarettes per day was not an independent risk factor for MetS in either gender.^30^

The relationship between serum UA levels and MetS has been well demonstrated in the literature. According to numerous studies, there is a positive association between serum UA and the prevalence of MetS. Furthermore, serum UA level was elevated significantly as the number of metabolic components increased.^4^, ^6^, ^31^, ^32^, ^33^ A higher level of serum UA is also an independent and strong factor for developing and predicting MetS in healthy middle‐aged population of both gender.^34^, ^35^ In subjects with elevated serum UA levels, the risk of incident type 2 DM is also increased.^36^, ^37^ In Iran, it has been reported that those with MetS have higher serum UA levels compared to those without MetS.^33^, ^38^ Safiri et al. reported that the association of serum UA with some components of MetS may indicate that serum UA might be an additional component of MetS in adolescents.^38^ Our results are confirmatory to previous findings. In our study, serum UA level was significantly higher in those with MetS; moreover, serum UA increased as the number of criteria (severity) of MetS increased.

HDL has anti‐inflammatory, antioxidant and atheroprotective properties with mitigates endothelial dysfunction through reverse cholesterol transport.^39^ HDL functionality has been reported to be negatively and independently associated with the risk of developing CVD.^12^ Our study showed that people with low HDL level were more likely to develop MetS than with normal HDL cholesterol, which is consistent with previous studies.^40^, ^41^ Furthermore, mean level of HDL significantly decreased as the number of components (severity) of MetS increase. As mentioned before, UHR is a novel inflammatory and metabolic marker, which increases in inflammatory conditions.^13^ The diagnostic role of UHR was first described by Kocak et al. in patients with MetS.^15^ Then, an increased UHR levels has been discussed in other metabolic and inflammatory conditions. Zhang et al. showed that UHR was an independent risk factor of non‐alcoholic fatty liver disease (NAFLD) among lean adults population who had normal range of HDL and UA.^42^ Conformingly, Zhu et al. demonstrated the association between UHR and onset of NAFLD in non‐obese Chinese population with normal lipid profile, in which higher UHR values were independently associated with increasing risk of NAFLD occurrence.^43^ In a retrospective cross‐sectional cohort study of 535 subjects by Aktas et al., a Higher level of UHR in hypertensive patients with poor‐controlled blood pressure (considered as SBP ≥140 or DBP ≥90 mmHg) was observed compared to those with well‐controlled blood pressure and healthy subjects. Moreover, UHR had considerably high sensitivity and specificity in detecting HTN patients with poor‐controlled blood pressure and it was correlated with both SBP and DBP, as well as serum creatinine, estimated glomerular filtration rate (eGFR), TG and BMI.^44^ Mansiroglu et al. reported that UHR was significantly elevated in patients with coronary fistula compared to the control group. Moreover, end diastolic measurement of the right ventricle was significantly higher in patients with fistula.^45^

In a study on 4551 patients diagnosed with type 2 DM in Shanghai, a positive correlation between UHR and diabetic macrovascular and microvascular complications such as CVD and chronic kidney disease (CKD) was observed in men and postmenopausal women, which may show the importance of measuring and lowering UHR level in a timely manner to prevent diabetic nephropathy. However, no correlation was observed between the prevalence of diabetic retinopathy and UHR level.^46^

The relationship between UHR and MetS has been investigated in few studies before. Kocak et al. pointed out that UHR could predict MetS better than all five MetS criteria and could use as a new a inflammatory marker.^15^ Then, Aktas et al. reported that UHR was significantly and positively associated with fasting plasma glucose (FPG) and HbA_1_c in men with type2 DM, so the study suggested that UHR could serve as a promising predictor of diabetic control.^14^ A study conducted on 817 people in 2021, reported that UHR is useful in diagnosis of MetS and can also be used to screen subjects at risk of MetS.^40^ However, in that study, the relationship between UHR and MetS severity was not evaluated. In the present study, we found a significant positive association between UHR and MetS. Furthermore, we identified that the mean levels of UHR in subgroups of the MetS (+) group increased with increasing MetS components, indicating that patients with MetS generally have higher serum UA level and lower HDL cholesterol level. This study is the first clinical study, which demonstrates a significant positive association between UHR and MetS severity. Another strength of present study is the large population included. One of the limitations of the present study is its retrospective design, which could cause selection bias. We also did not exclude patients with comorbidities such as DM. Nevertheless, we suggest conducting prospective studies with other statistical methods to assess the association between UHR and MetS, in future.

## 5 CONCLUSION

The present study showed that among Iranian adult population, there is a significant positive correlation between UHR level as a novel easy‐to‐assess inflammatory and metabolic indicator and MetS. Furthermore, there is a positive association between UHR and MetS severity.

## AUTHOR CONTRIBUTIONS

**Rana Kolahi Ahari:** Conceptualization (equal); writing – original draft (equal). **Amin Mansoori:** Conceptualization (equal); formal analysis (equal). **Toktam Sahranavard:** Writing – review and editing (equal). **Monireh Sadat Miri:** Writing – original draft (equal). **Sara Feizi:** Writing – original draft (equal). **Majid Ghayour‐Mobarhan:** Project administration (equal).

## FUNDING INFORMATION

This research did not receive any specific grant from funding agencies in the public, commercial or not‐for‐profit sectors.

## CONFLICT OF INTEREST STATEMENT

Not applicable.

## ETHICS STATEMENT

All participants included in the study were informed about the study and signed a written informed consent form before inclusion. The study was approved by the Ethic Committee of Mashhad University of Medical Sciences.

## Data Availability

The data that support the findings of our study are available from the corresponding author upon reasonable request. The data are not publicly available due to ethical or privacy restrictions.
